# Effects of repeated use of post-exercise infrared sauna on neuromuscular performance and muscle hypertrophy

**DOI:** 10.3389/fspor.2025.1462901

**Published:** 2025-03-04

**Authors:** Essi K. Ahokas, Helen G. Hanstock, Heikki Kyröläinen, Johanna K. Ihalainen

**Affiliations:** ^1^ Faculty of Sport and Health Sciences, University of Jyväskylä, Jyväskylä, Finland; ^2^ Swedish Winter Sports Research Centre, Department of Health Sciences, Mid Sweden University, Östersund, Sweden; ^3^ Finnish Institute of High Performance Sport KIHU, Jyväskylä, Finland

**Keywords:** heat therapy, recovery, physical performance, hypertrophy, team-sport, female athletes

## Abstract

**Purpose:**

The aim of this study was to investigate whether regular use of infrared sauna (IRS) after training can promote neuromuscular performance and positive changes in body composition during a 6-week training period.

**Methods:**

Forty female team sport athletes were pair-matched into two groups: IRS (*n* = 20) and control (CON; *n* = 20). Physical performance tests, body composition and hypertrophy measurements (dual-energy x-ray absorptiometry and ultrasound of m. vastus lateralis) were performed PRE and POST a 6-week strength and power training period, involving 2–3 sessions per week. Performance tests included a 20 m sprint, squat jump (SJ), countermovement jumps with body weight (CMJ) as well as with 15, 25, and 50% additional load (CMJ15%, CMJ25%, and CMJ50%), and a maximal isometric leg press (MVC). Participants in the IRS-group used IRS (10 min, 50℃) after training three times per week.

**Results:**

Training improved neuromuscular performance and muscle hypertrophy in both groups (*p* < 0.05). Following the discovery of an interaction effect for CMJ15% height (*p* = 0.002) and peak power (*p* = 0.010), *post hoc* tests revealed higher jump height POST-IRS (*p* = 0.006) and PRE-CON (*p* = 0.023) compared to PRE-IRS, and higher peak power POST-IRS (*p* = 0.002) compared to PRE-IRS. Furthermore, an interaction effect was observed for 5 m split time of the 20 m sprint (*p* = 0.020), but no differences were found between groups and timepoints. There were no interactions for the hypertrophy measures.

**Conclusion:**

Incorporating post-exercise IRS bathing does not significantly impact hypertrophy gains, but might boost long-term power production capabilities.

## 1 Introduction

Sauna bathing has become popular among athletes, not only for heat acclimation and rapid weight loss, but also to promote post-exercise recovery (1). Meta-analytical data show that post-exercise heat relieves pain experienced after exercise loading (2). However, traditional sauna bathing might be detrimental to next-day maximal physical performance (3). These detrimental effects have not been reported when post-exercise infrared sauna (IRS) has been used; on the contrary, post-exercise IRS has been shown to improve recovery of jump performance (4, 5). Whereas traditional saunas heat the air to approximately 70°C–100°C, which then heats the occupant primarily by convection, IRS instead radiate heat, while maintaining a lower air temperature of approximately 40°C–60°C. Even though radiated heat penetrates more deeply and causes occupants to sweat more vigorously at lower temperatures than in traditional saunas, IRS might provide a more comfortable and relaxing experience (6). Additionally, the cardiovascular responses are milder during IRS compared to traditional saunas (5).

The beneficial effects of infrared radiation during recovery may be attributed to increased peripheral blood flow (7), which could accelerate the clearance of edema, limit inflammation, and improve muscle repair (8). However, there have been conflicting results regarding inflammation and heat exposures. Local short-wave diathermy heat therapy was found to reduce intramuscular interleukin-6 (IL-6) and attenuate increases in intramuscular tumor necrosis factor-α levels after exercise (9), whereas passive whole-body heating increased plasma IL-6 and interleukin 1 receptor antagonist (10, 11). Furthermore, an animal study found that heat treatment accelerates inflammation reactions and enhances muscle regeneration after crush injury (12), which, however, is a much more potent stimulus compared to recovery from muscular exercise in humans. Nevertheless, speculated reduction of inflammation might be detrimental if the recovery method is used regularly, as has been reported with non-steroidal anti-inflammatory drugs [NSAIDs, (13)], which can negatively affect strength training adaptations. On the other hand, increasing muscle temperature increases the expression of heat-shock proteins (HSP) and activates the mTOR signaling cascade, which in turn promotes muscle growth (14).

The effects of regular use of post-exercise traditional sauna or IRS on strength training adaptations have not been studied. Nonetheless, heat exposure via regular hot-water immersion (HWI) during a four-week training period (including sport specific training, repeated sprints and strength training) improved isometric maximal strength in elite short-track speed skaters compared to passive recovery (15). However, there were no differences in squat jump (SJ) and counter-movement jump (CMJ) performance between HWI and control (15). In addition, long-term heat exposure without exercise training can induce muscle hypertrophy (16); meta-analytical data indicate that muscle mass increases in animal samples after passive heating (17), but in human studies, passive heating has been associated with both increased fiber hypertrophy (16) and no significant changes in hypertrophy (18). Moreover, regular use of post-exercise heat methods does not appear to influence athletes' lean mass (15, 19). Although 3 weeks' regular post-exercise sauna bathing in extreme heat (100°C) has been reported to reduce body mass and fat mass (19), most studies investigating the regular use of traditional saunas (20, 21) or HWI (15) have found no additional benefits of heat exposure on body mass or fat mass.

The aim of this study was to examine effects of regular use of post-exercise IRS on strength training adaptations in female team sport athletes, including assessment of neuromuscular performance and body composition.

## 2 Methods

### 2.1 Participants

Forty female team sport athletes participated in the study. Teams were recruited from local basketball (two teams, *n* = 14), futsal (two teams, *n* = 18), ice hockey (*n* = 4) and American football clubs (*n* = 4). Performance level ranged from the highest league (*n* = 12) to 3rd highest leagues (*n* = 4), with the majority of teams competing in the second tier in Finland. Athletes were pair-matched into two groups, an IRS group (IRS, *n* = 20, age: 22 ± 5 years; height: 167 ± 7 cm; body mass: 69.4 ± 18.2 kg) and a control group (CON, *n* = 20, age: 23 ± 5 years; height: 168 ± 6 cm; body mass: 69.5 ± 13.7 kg), with the matching process prioritizing team affiliation, followed by neuromuscular performance [20 m sprint, CMJ, maximal voluntary contraction (MVC)], and age. Groups were evenly allocated within teams.

Prior to inclusion, participants were informed about the study purposes and test procedures, and written informed consent was obtained from all participants. For participants who were under the age of 18 years, written consent from a guardian was also obtained. In addition, participants completed a health screening questionnaire. This study was conducted according to the Declaration of Helsinki (2013), except for registration in a database, and ethical approval was provided by the Ethics Committee of the University of Jyväskylä (1516/13.00.04.00/2021).

Previous sauna use was evaluated with a questionnaire about sauna bathing. Three participants (IRS: 1, CON: 2) reported that they had not used a sauna during the past six months. 21 (IRS: 11, CON: 10) reported that they had sauna bathed about once a month, and 8 (IRS: 5, CON: 3) once a week. Six (IRS: 3, CON: 3) participants reported that they had sauna bathed 2–5 times a week. A participant had been in an IRS once, while the others had never used them. Two participants did not return the completed questionnaire.

### 2.2 Study design

A pair-matched case-control design was used to examine whether regular IRS use could promote adaptations in neuromuscular performance and body composition during a 6-week training period. Athletes were recruited as participants, to ensure specificity of the data to the athletes of higher performance level, given physiological responses to exercise may differ between recreationally active participants and athletes (22). The frequency of strength training corresponded to the athletes' normal training with a frequency of 1–3 times per week, which may also increase the applicability of the study to performance sport (23). For the training intervention, a new stimulus in the form of strength- and power-based training was introduced. Consequently, the training volume and intensity were adjusted accordingly.

The experimental trials were completed before (PRE) and after (POST) the 6-week strength and power training period. All participants were instructed not to consume any alcohol, and to refrain from exercise for 24 h prior to the experimental trials and on the day of experimental trials. A fluid intake questionnaire was completed 24 h prior to the first experimental trial to ensure similar fluid intake prior to the POST measurements.

Physical performance tests and resting measurements were conducted during experimental trials. PRE and POST resting measurements took place at the same time of day (6:00–9:30 a.m.) after 10 h of fasting. These measurements included ultrasound imaging of the vastus lateralis muscle and body composition by dual-energy x-ray absorptiometry (DXA). Performance tests were carried out in the afternoon or early evening. They included 20 m sprint, squat jump (SJ), and CMJ tests with body weight and with additional loads, and isometric leg press.

Strength and power training was performed 2–3 times per week and sauna bathing in IRS (10 min, 50℃) was carried out three times per week. Each participant completed a menstrual and training diary between the PRE and POST measurements. All participants were instructed to keep their diet consistent during the intervention period.

### 2.3 Procedures

#### 2.3.1 Training program

Strength and power training sessions were performed twice (two teams, *n* = 10) or three times (four teams, *n* = 30) per week. A standardized warm-up was performed at the beginning of each session (∼10 min). The exercises were selected based on the physical demands of the participants' respective sports. In addition, the exercises were performed in pairs by combining a resistance exercise with an explosive exercise. Most exercises were performed as quickly as possible. During the first and second week, three sets were performed, and the weights were 70%–80% of assessed one repetition maximum (1RM). During the third and fourth week, four sets were performed, and the weights were 80%–85% of assessed 1RM. The load was decreased during the fifth and sixth week (3 sets, 50%–55% 1RM). The repetitions in reserve (RIR) were used to optimize the predetermined relative weights (24). This approach allows for adjustments based on how many repetitions a participant estimates they could still complete at the end of a set. This ensures that the load is challenging yet manageable to optimize training outcomes (25). The exercises and the repetitions are shown in Table 1. The rest periods used between the sets and exercises were 2 min.

**Table 1:** Exercises and repetitions (reps) of the training program's weekly sessions.

Session	Exercise	Reps
1	1.1 Squats	4
	1.2 Box jumps	6
	2.1 Land mine curtsy lunges	4 + 4
	2.2 Drop jumps	6
	3.1 Pull ups/Lat pull down	4
	3.2 Medicine ball slams	6
2	1.1 Hip thrusts	4
	1.2 10 m resisted sprint	4
	2.1 Forward lunges	4 + 4
	2.2 Hop run	6 + 6
	3.1 Bench press	4
	3.2 Medicine ball throw	6
3	1.1 Split squats	4
	1.2 Drop jumps	4
	2.1 Hip thrusts	4
	2.2 Horizontal jumps	4
	3.1 Military press	4
	3.2 Trunk rotation with bar	6

If only two sessions were performed training sessions 1 and 2 were utilized.

Aside from the prescribed training intervention, athletes otherwise trained according to their team's own training program (sport-specific and endurance training). Participants were instructed to avoid using other recovery strategies (foam rolling, massage, compression clothing, NSAIDs) aside from the intervention assigned to each group in the present study.

#### 2.3.2 Infrared sauna sessions

Participants in the IRS group sat in the IRS for 10 min, wearing sports bras and shorts, three times per week (18 occasions in total). Sauna bathing in IRS was done after strength exercise sessions or sport-specific training sessions. The temperature of the sauna was set to 50°C 15–25 min before sauna bathing, but the measured air temperature at the level of the hips was 31.3 (4.6)°C, with 48 (10) % relative humidity (RH). Two different full spectrum IRS models were used. The first (VitaMy, Sentiotec GmbH, Vöcklabruck, Austria) had two seats with IR-emitting panels at the front and back. The second sauna (Harvia Spectrum Small SGS1310, Harvia Plc, Muurame, Finland) had two seats with IR-emitting panels at the back and side. Seven participants used the first IRS, ten utilized the second IRS, and three used both.

The body temperature of the participants during sauna bathing was not measured but was piloted with eight participants [age 30.4 (3.5) years, height 168.3 (6.5) cm, body mass 62.6 (6.1) kg]. Tympanic temperature (T_tymp_) was measured with Braun ThermoScan PRO 600 (Braun GmbH, Kronberg, Germany) and skin temperature (T_sk_) was recorded using FORA IR10 Multi-Temp thermometer (ForaCare Suisse AG, St. Gallen, Switzerland) from four sites on the left side of the body: m. pectoralis major, m. biceps brachii, m. rectus femoris, and m. gastrocnemius lateral head. The measurements were taken immediately before and after the sauna. T_sk_ was calculated as a weighted mean according to Ramanathan (26). T_tymp_ increased from 36.7 (0.3)°C and 36.5 (0.3)°C to 37.2 (0.3)°C and 37.1 (0.2)°C in Harvia Spectrum Small and VitaMy, respectively. Weighted mean T_sk_ increased from 34.3 (1.4)°C and 33.3 (1.1)°C to 36.5 (0.1)°C and 36.4 (0.2)°C. The measured air temperature was 33.1 (3.1)°C and 29.0 (1.9)°C in Harvia Spectrum Small and VitaMy, respectively.

#### 2.3.3 Performance measurements

In the 20 m sprint test, 5 m and 20 m split times were recorded with photocells (University of Jyväskylä, Finland). The participants had three attempts to achieve their best sprint performance, with a 3-min rest between trials, and the best 5 m and 20 m times reported. Previous studies have reported a high intraclass correlation coefficient (ICC; 0.93–0.98) of the 20 m sprint test (27, 28).

The jump height (cm) of SJ and CMJ was calculated from the measured take-off velocity with a force plate (FP8, Hur Labs Oy, Kokkola, Finland). Peak power (W) of the jumps was also analyzed from force signals of the force plate. SJ and CMJ were performed without additional load. CMJ tests were also performed with additional loads: 15% (CMJ15%), 25% (CMJ25%), and 50% (CMJ50%) of body mass, which was measured with a digital scale (SECA GmbH, Hamburg, Germany) immediately before the performance testing. During the SJ- and CMJ-tests without additional load, participants were asked to keep their hands on their hips and jump as high as possible. CMJ with additional loads were performed with a barbell held on shoulders and weight plates. Participants had three attempts to achieve their best performance in each jump test with 1 min rest between trials. A self-determined range of motion was allowed. A previous study has reported the reliability of SJ- and CMJ- tests, with ICC of 0.97 and 0.98, and coefficients of variation (CV) of 3.3% and 2.8%, respectively (29).

The bilateral isometric leg press test served as a measure of maximal voluntary contraction (MVC) and was measured on a leg press dynamometer (University of Jyväskylä, Finland). During the test, the knee angle was 107°. The participants had three attempts to achieve their best performance with 1 min rest between trials. A previous study has reported high ICC (0.98) and low CV (3.48%) values for an isometric leg press test (30).

#### 2.3.4 Body composition and hypertrophy measurements

Body composition was measured by Dual-energy x-ray absorptiometry (DXA; Lunar Prodigy Advance, GE Medical Systems -Lunar, Madison WI USA). A whole-body scan was performed, and delineation of the lower body was reviewed manually. A previous study has reported the reliability of body mass, fat mass, and lean mass with an ICC of 0.999, 0.998, and 0.995 and CV of 2.3%, 1.6%, and 0.3%, respectively for whole-body measurements (31).

Morphological parameters of the m. vastus lateralis of the right leg were determined from images obtained using a diagnostic ultrasound system (Aloka alpha10, Japan). At PRE, the midpoint between the greater trochanter and the superior aspects of the patella were measured. A straight line in parallel with the transverse axis of the thigh was extended from the midpoint that had been marked. The probe was placed above the line (transversally to the longitudinal axis of the thigh) to gain the cross-sectional area (CSA) image. To obtain the pennation angle image, the probe was placed in parallel with the longitudinal axis of the thigh. The distance between the probe for the pennation angle image and the marked midpoint was recorded for future scanning (POST), and participants were recommended to reinforce the markings with permanent marker. Ultrasound images were analyzed with ImageJ (National Institutes of Health, Maryland, United States). In previous studies, ICC of m. vastus lateralis' CSA and pennation angle were 0.969–0.988 and 0.85–1.00, respectively (32, 33).

#### 2.3.5 Training and menstrual diaries

Participants completed a training diary (training types, frequency, volume, session perceived exertion) from the PRE to POST-tests. The training load was calculated as the product of session volume (h) and session perceived exertion (5-point scale).

A menstrual diary was also completed between PRE and POST. Participants reported days of bleeding, possible symptoms, and the use of nonsteroidal anti-inflammatory drugs. Furthermore, the diary included questions about the regularity of the menstrual cycle, the use of hormonal contraceptives and dates of the previous menstruation. The cycle was divided in the first and the second half of the cycle based on the length of the cycle and the assumption that the duration of the luteal phase is about 14 days (34). 23 participants (IRS: *n* = 9; CON: *n* = 14) were naturally menstruating. However, there were 4 amenorrheic or oligomenorrheic participants (IRS: *n* = 3; CON: *n* = 1) among the naturally menstruating participants. 7 participants (IRS: *n* = 3; CON: *n* = 4) used combined hormonal contraceptives and 5 participants (IRS: *n* = 4; CON: *n* = 1) used progestin-only hormonal contraceptives. Five athletes (IRS, *n* = 4; CON, *n* = 1) did not return the diary. The use of hormonal contraceptives, manifestation of amenorrhea, oligomenorrhea (among naturally menstruating participants), or irregular cycles (among progestin-only contraceptive users), as well as cycle phase during the experimental trials are presented in Supplementary Table S1.

### 2.4 Statistical analyses

Statistical analyses were performed using IBM SPSS Statistics 28.0 (SPSS Inc., Chicago, IL). A generalized estimating equation (GEE) approach with an unstructured working correlation matrix and a linear model was used to analyze main (group and time) and interaction (time × group) effects, utilizing data from the IRS and CON groups. The GEE model utilizes information also from incomplete observations and considers correlations between outcomes across time for the same participant. In model 1, only main effects were analysed. In model 2, both main and interaction effects were used. Model 2 outcomes were presented only if the interaction effect was statistically significant. Significance was set at *p* < 0.05.

Following GEE-models, paired and independent samples *t*-tests were used to evaluate main and interaction effects. Normal distribution of the data was assessed both visually and using Shapiro–Wilk's test. Independent samples *t*-tests were used to analyze the difference between groups at PRE and POST. Within group changes were evaluated with paired samples *t*-tests. Non-normally distributed data were analyzed with Wilcoxon signed-rank test and Mann–Whitney *U*-test. Data were presented using mean and standard deviation (SD).

Two participants dropped out during the intervention. One additional participant did not follow the instructions and used traditional sauna and HWI in her free time. The results of these three participants and a case-control pair of one drop-out were excluded from further statistical analyses. The analyses were therefore conducted with 36 participants (IRS: *n* = 18, CON: *n* = 18).

## 3 Results

### 3.1 Training volume, load, and frequency

Total training volume (*p* = 0.860) and load (*p* = 0.708) did not differ between the groups. In addition, the training frequency of strength and power training sessions were similar in both groups (*p* = 0.286; Table 2).

**Table 2:** Mean (SD) training volume and load during the intervention, and number of strength and power training (RT) sessions.

TRAINING	IRS	CON
Total volume (h)	38.5 (12.2)	37.8 (11.3)
Volume of endurance training (h)	3.8 (4.5)	3.2 (4.1)
Volume of sport specific training (h)	15.8 (14.7)	16.6 (13.9)
Volume of strength training (h)	19.9 (4.4)	18.9 (3.3)
Total load	108.5 (40.5)	114.3 (49.1)
Number of RT sessions	16.8 (3.9)	15.6 (2.7)

The load was calculated as the product of session volume and session perceived exertion.

### 3.2 Physical performance

There were no time, group, or interaction effects for 20 m sprint, MVC, height of SJ, CMJ, CMJ25%, and CMJ50%, and peak power during CMJ50% (Supplementary Table S2).

**Figure 1:**
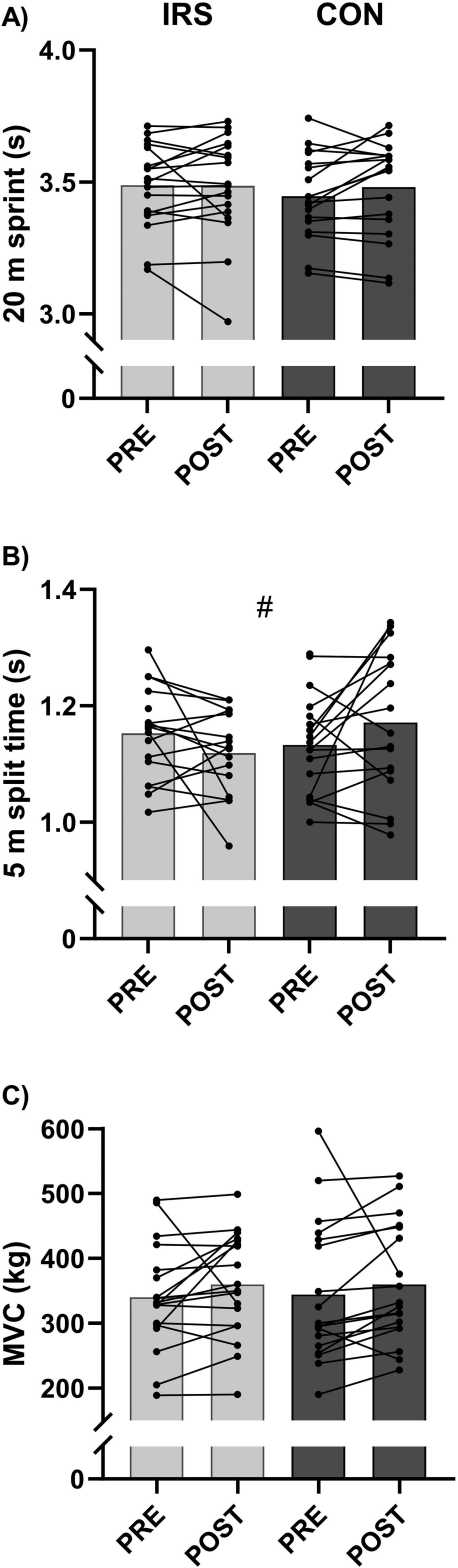
Mean and individual values of **(A)** 20 m sprint, **(B)** 5 m split time, and **(C)** isometric leg press (MVC). CON, control group; IRS, infrared sauna group; PRE, before the intervention; POST, after the intervention. # Indicates interaction effect between IRS and CON in GEE-model (*p* < 0.05).

A time × group interaction effect was observed for 5 m split time (B = −0.075, *p* = 0.020; Figure 1, Supplementary Table S2). However, paired sample *t*-tests revealed only a trend toward slower split times POST-CON [1.171 (0.123) s] vs. PRE-CON [1.133 (0.085) s, *p* = 0.083]. In IRS, faster, but not statistically significant 5 m split times (*p* = 0.171) were found at the POST- compared to the PRE-test. Furthermore, 5 m split times were different between groups (B = 0.095, *p* = 0.049) in model 2.

**Figure 2:**
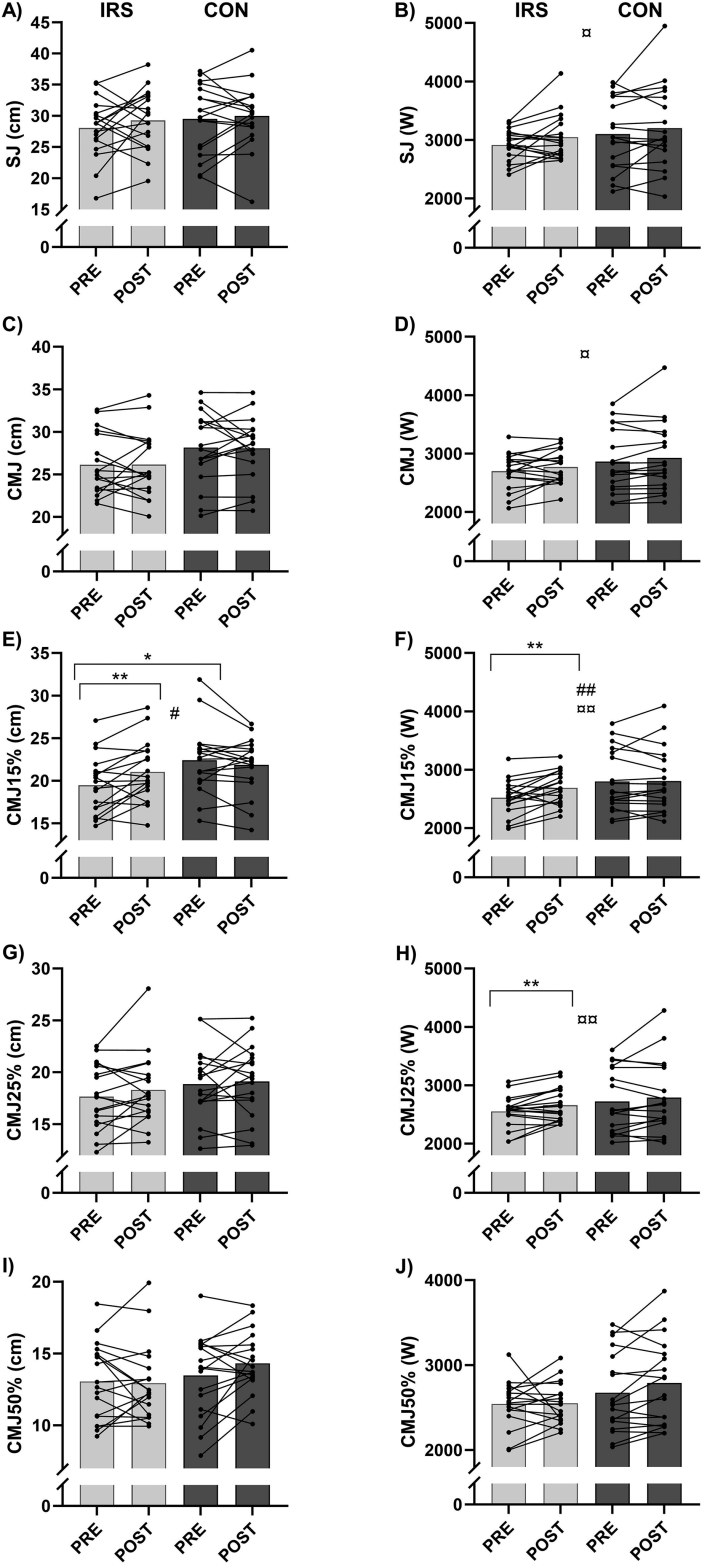
Mean and individual values of jump test results. **(A)** squat jump (SJ) height, **(B)** squat jump (SJ) peak power, **(C)** countermovement jump (CMJ) height, **(D)** countermovement jump (CMJ) peak power, **(E)** countermovement jump with 15% additional load (CMJ15%) height, **(F)** countermovement jump with 15% additional load (CMJ15%) peak power, **(G)** countermovement jump with 25% additional load (CMJ25%) height, **(H)** countermovement jump with 25% additional load (CMJ25%) peak power, **(I)** countermovement jump with 50% additional load (CMJ50%) height, and **(J)** countermovement jump with 50% additional load (CMJ50%) peak power. CON, control group; IRS, infrared sauna group; PRE, before the intervention; POST, after the intervention. *Indicates differences in *t*-tests (*p* < 0.05); **indicates differences in *t*-tests (*p* < 0.01); # indicates interaction effect between IRS and CON in GEE-model (*p* < 0.05); ## indicates interaction effect between IRS and CON in GEE-model (*p* < 0.01), ¤ indicates time effect in GEE-model (*p* < 0.05), ¤¤ indicates time effect in GEE-model (*p* < 0.01).

There were time × group interaction effects (*p* = 0.002–0.010) for CMJ15% jump height and peak power in model 2. For jump height, paired and independent sample *t*-tests revealed a difference between PRE-IRS [19.5 (3.4) cm] and POST-IRS [21.0 (3.6) cm, *p* = 0.006], as well as between PRE-IRS and PRE-CON [22.4 (4.0) cm, *p* = 0.023; Figure 2]. For CMJ15% peak power, a difference was found between the PRE-IRS [2,519 (299) W] and POST-IRS [2,690 (284) W, *p* = 0.002]. In addition, there was a trend towards a significant difference between PRE-IRS and PRE-CON [2,799 (528) W, *p* = 0.061]. Furthermore, there was a time effect (B = 88.3, *p* = 0.011) for CMJ15% peak power in model 1 and group effect (*p* = 0.002–0.007) for CMJ15% jump height and peak power in model 2.

There were time effects for peak power achieved in SJ (B = 119.4, *p* = 0.021), CMJ (B = 65.8, *p* = 0.028), and CMJ25% (B = 87.3, *p* = 0.006) in model 1. However, there were no differences in paired and independent sample *t*-tests for SJ and CMJ, but CMJ25% peak power improved in the IRS-group from PRE [2,551 (275) W] to POST W, *p* = 0.008).

### 3.3 Body composition and hypertrophy

**Figure 3:**
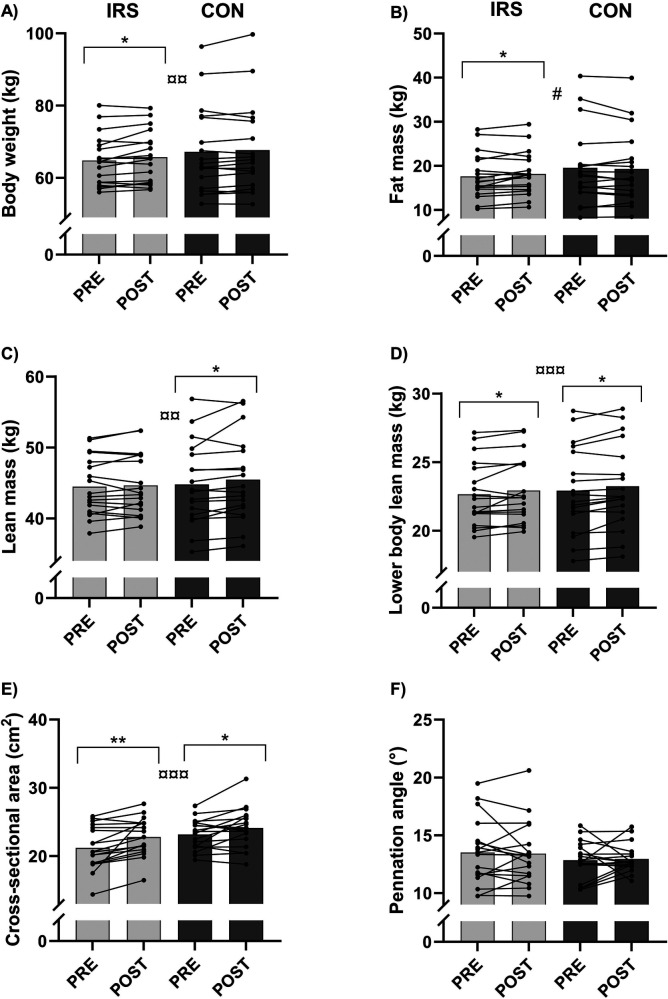
Mean and individual values of **(A)** body mass, **(B)** fat mass, **(C)** lean mass, **(D)** lower body lean mass, **(E)** cross-sectional area of vastus lateralis, and **(F)** pennation angle of vastus lateralis. CON, control group; IRS, infrared sauna group; PRE, before the intervention; POST, after the intervention. *Indicates differences in *t*-tests (*p* < 0.05); **indicates differences in *t*-tests (*p* < 0.01); # indicates interaction effect between IRS and CON in GEE-model (*p* < 0.05), ¤¤ indicates time effect in GEE-model (*p* < 0.01), ¤¤¤ indicates time effect in GEE-model (*p* < 0.001).

There was no interaction effect for body mass (Supplementary Table S3). However, a time effect (B = 0.69, *p* = 0.003) was observed for body mass which, based on the paired *t*-tests, increased only in IRS [PRE: 64.8 (7.1) kg, POST: 65.7 (7.2) kg, *p* = 0.028]. Furthermore, there was time × group interaction effect (B = −0.81, *p* = 0.036) for fat mass in model 2. Paired samples *t*-tests revealed difference between the PRE- [17.60 (5.10) kg] and POST-values in IRS [18.2 (4.9) kg; *p* = 0.048; Figure 3].

There was no interaction effect for lean mass, but a time effect (B = 0.43, *p* = 0.003) was found when lean mass increased in CON [PRE: 44.8 (5.8) kg, POST: 45.5 (6.0) kg, *p* = 0.050]. A time effect (B = 0.30, *p* < 0.001) was found for lower body lean mass, whereby mass increased in the IRS (*p* = 0.041) and CON groups (*p* = 0.011) [PRE: 22.7 (2.4) kg, 22.9 (3.2) kg; POST: 22.9 (2.4) kg, 23.2 (3.1) kg, respectively]. Furthermore, there was time effect (B = 1.28, *p* < 0.001) for the CSA of the m. vastus lateralis in model 1. Paired sample *t*-tests revealed an increase in CSA from PRE [IRS: 21.21 (3.15) cm^2^, CON: 23.17 (2.19) cm^2^] to POST [IRS: 22.81 (2.74) cm^2^, 24.12 (2.91) cm^2^] in both groups (IRS: *p* = 0.002, CON: *p* = 0.030). There were no time, group, or interaction effects for pennation angle of the m. vastus lateralis.

## 4 Discussion

The present study investigated whether regular use of post-exercise IRS could further enhance neuromuscular performance and improve body composition in female team-sport athletes. The main findings were that 6 weeks of training induced lower body hypertrophy, but the use of IRS did not contribute any additional changes in hypertrophy. However, IRS elicited improvements in power production capacity, which was observed in jump performance with additional weights and the 5 m split time of sprint.

In the neuromuscular performance tests, 20 m sprint time, MVC, and height attained on the SJ, CMJ, CMJ25%, and CMJ50% tests did not improve in IRS or CON following the training intervention. Peak power improved after the intervention in all the jump tests, except for CMJ50%. Notably, an interaction effect was observed for CMJ15% jump height and peak power, indicating differential improvements between the groups. In addition, when the results were analyzed within groups, changes were found only in the IRS-group, where peak power in both CMJ15% and CMJ25% improved. Although CMJ15% height was lower in PRE-IRS compared to PRE-CON, the 6-week post-exercise IRS intervention improved CMJ15%, whereas there was no change in CON. Previous studies have found that baseline performance levels do not affect training-induced adaptations (35, 36). Therefore, in this study, the difference in baseline levels in CMJ15% height based on the independent *t*-test is unlikely to explain the discrepancy in CMJ15% improvement during the training intervention. In addition, for the 5 m split time from the 20 m sprint, a time × group interaction was observed, indicating that the change over time differed between the groups. Specifically, only the CON group showed a trend toward slower times at POST compared to PRE, while other *post hoc* analyses did not revealed any differences. Taken together, these positive changes in the IRS group could have resulted from improved acute recovery (4), enabling more effective training, or triggering physiological responses from heat exposure that enhance training-induced neuromuscular adaptations (14). However, it is challenging to speculate why IRS supported performance improvements exclusively in one variant of loaded jumps. The training aimed to develop both strength and power characteristics by incorporating both resistance exercises and speed-oriented movements, such as jumps (37, 38). However, the loads of resistance exercises were chosen to focus more on improving power production than maximal strength production (39). Thus, the sets where not performed near to failure and participants were instructed to perform the exercises as fast as possible (38). While the intervention did not enhance maximal isometric force production or speed-related attributes, it may have improved the middle portion of the force-velocity curve when IRS exposure was included.

As the effects of regular post-exercise IRS on strength training adaptations have not been studied, it may be relevant to compare our findings with studies that have examined training adaptations following other post-exercise heat exposure methods. While the mechanism of heating differs; traditional saunas and HWI heat the occupant through convection of heated air or water, whereas IRS uses radiated heat; all whole-body heat exposures share the ability to increase body temperature and trigger physiological responses associated with elevated body temperature. However, hydrostatic pressure also affects the physiological response in HWI (40). Contradictory findings have been reported following regular HWI (3 times/week, 20 min at 40°C) over four weeks' training in short-track speed skaters, where isometric maximal strength increased compared to passive recovery, but there were no differences in jump test results between HWI and passive recovery (15). Furthermore, a heat acclimation protocol utilizing HWI (4 × 45 min at 40°C) and post-exercise traditional sauna bathing (4 × 45 min at 40°C and 80% RH) improved peak power output in repeated 6-second maximal cycle ergometer sprints among female rugby sevens players (41). However, no control group was used in this study. While these studies provide some insight into the effects of post-exercise heat exposure on training adaptations, the specific impact of heat exposures on strength training adaptations remains unclear. Although results to date have been mixed—possibly due to varying methodologies—most studies report at least some positive effects of heat exposure on strength and power output. This suggests potential benefits of post-exercise heat exposure, even if the mechanisms and optimal parameters remain uncertain. Future research is essential to further elucidate these findings and to explore how IRS compares to other heat modalities.

The use of local heat stress with and without exercise training has also been studied, offering valuable insights into how elevating muscle temperature may influence the development of force production capacity. Local heating combined with strength training has shown benefits for strength adaptations: a 10-week low-intensity elbow flexion program (3 × 30 reps, <30RM) with 60 min of local heating before and during exercise improved maximum isometric torque (42), while a 6-week elbow extension program (3 × 8 reps, 30RM) with 20 min of pre-heating enhanced 1RM in untrained participants (43). Passive heat without exercise also enhanced strength: heat- and steam-sheets (10 weeks, 8 h/day, 4 days/week) or heat garment (∼52°C, 8 weeks, 90 min/day, 5 days/week) boosted maximal isometric force (16), and torque (18), respectively. Furthermore, passive whole-body heat stress in an environmental chamber (11 days, 1 h/day, 48°C–50°C and 50% RH) improved maximal voluntary contraction and muscle contractile function in non-strength-trained individuals (44). In the present study, the use of heat, however, did not affect maximal force production measured via isometric leg press. Passive heat appears to enhance force production in physically inactive individuals (16, 18) or those not engaged in strength training (44). In contrast, post-exercise heat does not improve maximal force production in athletes already undergoing extensive sport-specific training, including strength and conditioning training, as observed in the present study. It should be also noted, that all the studies related to local and passive heating used young (∼20–45 years) male participants, except the Racinais et al. (44), which included both women and men.

Body composition is widely recognized as a key factor influencing athletic performance (45). In this study, body mass and fat mass increased in the IRS group, whereas lean mass increased in the CON group. Both groups experienced increases in lower body lean mass and the CSA of the m. vastus lateralis. This likely reflects the focus of the strength and power training on the lower body. The findings suggest that regular IRS use after exercise does not enhance hypertrophy, aligning with literature showing no hypertrophic effects of post-exercise HWI (15) or sauna bathing (19). Interestingly, greater increases in CSA of the knee extensors and flexors have been observed in passive recovery compared to HWI (15). Conversely, local pre-heating or heating before and during training has been associated with increased CSA in the elbow flexors (42) or extensors (43), and long-term passive heating (e.g., 8 h/day) has been shown to increase quadriceps CSA and mean fiber CSA (16), whereas shorter durations passive and local heating (e.g., 90 min/day) did not yield changes (18). Additionally, conflicting results exist regarding body mass and fat mass, such as sauna bathing being linked to reductions in body mass and fat mass (19), while other studies found post-exercise heat did not significantly affect body mass or fat mass compared to controls (15, 20, 21), consistent with the present findings.

Incorporating heat after exercise seems overall less effective than passive heating for improving muscle strength, power, and hypertrophy. Notably, post-exercise whole-body heating methods are generally applied for shorter durations. For instance, HWI and sauna protocols often last 20–40 min, 3–4 times per week (15, 19), whereas local heating interventions can extend up to 8 h (16). Although post-exercise heating methods may use higher temperatures (19–21), they may result in lower peak muscle temperatures or shorter durations of elevated temperature compared to passive heating. For example, 8-h local heating raised muscle temperature from 34.9°C to 38.2°C within 3 h and maintained it at 38.3°C for an additional 3 h. Heat shock proteins (HSPs), not analyzed in this study, may mediate the positive effects of heating by protecting cells, enhancing muscle regeneration, and promoting hypertrophy (14, 46). HSP expression, associated with increased muscle fiber CSA in rats (47), is highly dependent on elevated muscle temperature (14, 48). Evidence suggests that muscle temperatures of 38°C (17) or higher, up to 40°C (14), are required to induce beneficial effects on hypertrophy. In addition to HSPs, mammalian target of rapamycin (mTOR) kinases may also mediate the positive effects of heating by stimulating cell growth when activated (14, 49). For instance, 30 min of HWI at 41°C increased Akt and p70S6K phosphorylation in the soleus and plantaris muscles of rats (50). Heating may also influence gene expression related to muscle hypertrophy and atrophy, as shown in the *in vitro* differentiation of C2C12 myogenic cells (51). Thus, 10 min in IRS might have been too brief to sufficiently raise muscle temperature. Previously, a 10-min post-exercise HWI (40°C) was found to elevate muscle temperature compared to baseline and passive recovery (52). Nevertheless, the temperature immediately after the HWI at a depth of 3 cm was only 37.2 (0.3)°C (52). The 10 min IRS protocol used in the present study was designed primarily to be practical, feasible, and safe for team sport athletes. However, it is likely that the duration did not support heat-induced physiological responses favourable for hypertrophy but may have contributed to the acute recovery from training sessions. It should be noted that the whole body heat methods could pose additional stress on athletes (53), increasing sympathetic nervous system activity (54) and energy expenditure (55), and potentially impairing sleep and recovery for subsequent training sessions (56). Therefore, the duration of post-exercise heat exposure should be carefully tailored, balancing potential benefits with practical considerations and recovery needs.

One strength of this study is the inclusion of athletes as participants, acknowledging that physiological responses to exercise can differ significantly between competitive athletes and recreationally active individuals (22). The strength exercise training performed by the athletes closely mirrored their usual routines, enhancing the study's relevance to high-performance sports contexts (41). However, trained athletes often exhibit reduced potential for noticeable adaptations over short training periods, likely due to their already high baseline training status (23). This might explain the modest physical performance improvements observed in athletes despite increases in muscle mass. Furthermore, the strength of this study would have been increased if the muscular temperature of the participants had been measured during and after the recovery protocol.

According to current research, it seems unlikely that menstrual cycle phase would have an effect on strength performance or hypertrophic adaptations on a group level (57). However, there might be individual variability and there are not enough high-quality studies regarding this issue (57). In addition, in the present study almost half of the participants performed the PRE and POST-tests in the same phase of menstrual or hormonal cycle. Furthermore, the tests of the remaining were distributed across different phases. However, random sampling of females across hormonal contraceptive use and menstrual cycle phase could provide more ecologically valid findings for female athletes (58). Even though measuring physical performance during the same cycle phase would have been the most optimal for reproducibility, it would have been practically challenging, as the duration of the training intervention would have varied between individuals due to differences in the length of their cycles.

In conclusion, the regular use of brief 10-minute post-exercise IRS session does not further enhance training-induced improvements in neuromuscular performance, except jump performance with small additional weight and the acceleration performance in the initial meters of the sprint, and changes in body composition. Furthermore, the observed positive effects of regular IRS usage are either minor or unclear, warranting further research. In addition, our data give no reason to expect that brief IRS after exercise could impair training adaptations. Thus, if IRS accelerates recovery from an acute exercise loading or alleviates muscle soreness as observed in other studies, it could be a valuable tool in athletes' recovery strategies. Further studies should focus on determining whether there is an optimal heat load (temperature and duration) that could improve long-term physical performance and body composition without imposing excessive stress on athletes' autonomic nervous system, sleep, and recovery.

## Data Availability

The raw data supporting the conclusions of this article will be made available by the authors, without undue reservation.
