# Supplementary material for: Effects of repeated use of post-exercise infrared sauna on neuromuscular performance and muscle hypertrophy
Source: Front Sports Act Living. 2025 Mar 4;7:1462901. doi: 10.3389/fspor.2025.1462901 (PMC11913669; doi:10.3389/fspor.2025.1462901)
Supplement: Supplementary file 1 [file Table1.docx]

***Supplementary Material***

**Supplementary table 1.** The use of hormonal contraceptives, manifestation of amenorrhea, oligomenorrhea, and cycle phase during experimental trials.

|  | IRS | CON |
| --- | --- | --- |
| Naturally menstruating (NM) | 9 | 14 |
| Amenorrheic/ oligomenorrheic (NM only) | 3 | 1 |
| Combined hormonal contraceptives (CHC) | 3 | 4 |
| Progestin-only hormonal contraceptives (PHC) | 4 | 1 |
| PRE- and POST-tests in the same phase of the MC | NM = 4, CHC = 2 | NM = 5, CHC = 4 |
| PRE-tests in the 1^st^ and POST-tests in the 2^nd^ | NM = 1 | NM = 3 |
| PRE-tests in the 2^nd^ and POST-tests in the 1^st^ | NM = 1 | NM = 5 |
| PRE-tests in the ACT and POST-tests in the INACT | CHC = 0 | CHC = 0 |
| PRE-tests in the INACT and POST-tests in the ACT | CHC = 1 | CHC = 0 |

ACT = active phase; CON = control group; IRS = infrared sauna group; INACT = inactive phase; MC = menstrual cycle; PRE = before intervention; POST = after intervention.

**Supplementary table 2.** Changes in physical performance analyzed by general equation estimation.

|  |  | Model 1 | | | | | Model 2 | | | | |
| --- | --- | --- | --- | --- | --- | --- | --- | --- | --- | --- | --- |
|  |  | B | SE | 95 % CI Lower | 95 % CI Upper | p-value | B | SE | 95 % CI Lower | 95 % CI Upper | p-value |
| 20 m sprint | Group | 0.023 | 0.054 | -0.083 | 0.129 | 0.666 |  |  |  |  |  |
| (s) | Time | 0.016 | 0.016 | -0.014 | 0.047 | 0.297 |  |  |  |  |  |
|  | T*G interaction |  |  |  |  |  |  |  |  |  |  |
| 5 m split- | Group | -0.015 | 0.025 | -0.064 | 0.034 | 0.543 | **0.095** | **0.048** | **0.001** | **0.189** | **0.049** |
| time (s) | Time | 0.005 | 0.018 | -0.029 | 0.040 | 0.759 | 0.043 | 0.025 | -0.007 | 0.092 | 0.092 |
|  | T*G interaction |  |  |  |  |  | **-0.075** | **0.032** | **-0.139** | **-0.012** | **0.020** |
| MVC (kg) | Group | -2 | 28 | -57 | 52 | 0.891 |  |  |  |  |  |
|  | Time | 18 | 10 | -3 | 38 | 0.088 |  |  |  |  |  |
|  | T*G interaction |  |  |  |  |  |  |  |  |  |  |
| SJ height | Group | -1.07 | 1.50 | -4.00 | 1.86 | 0.473 |  |  |  |  |  |
| (cm) | Time | 0.84 | 0.68 | -0.50 | 2.17 | 0.219 |  |  |  |  |  |
|  | T*G interaction |  |  |  |  |  |  |  |  |  |  |
| SJ power | Group | -169.3 | 159.6 | -482.2 | 143.6 | 0.289 |  |  |  |  |  |
| (W) | Time | **119.4** | **51.7** | **18.1** | **220.6** | **0.021** |  |  |  |  |  |
|  | T*G interaction |  |  |  |  |  |  |  |  |  |  |
| CMJ height | Group | -1.96 | 1.18 | -4.29 | 0.36 | 0.097 |  |  |  |  |  |
| (cm) | Time | -0.05 | 0.39 | -0.80 | 0.71 | 0.905 |  |  |  |  |  |
|  | T*G interaction |  |  |  |  |  |  |  |  |  |  |
| CMJ power | Group | -158.6 | 142.9 | -438.7 | 121.5 | 0.267 |  |  |  |  |  |
| (W) | Time | **65.8** | **29.9** | **7.2** | **124.5** | **0.028** |  |  |  |  |  |
|  | T*G interaction |  |  |  |  |  |  |  |  |  |  |
| CMJ15% | Group | -1.87 | 1.11 | -4.04 | 0.30 | 0.009 | **-5.06** | **1.60** | **-8.20** | **-1.93** | **0.002** |
| height (cm) | Time | 0.51 | 0.38 | -0.25 | 1.26 | 0.187 | -0.56 | 0.48 | -1.49 | 0.38 | 0.242 |
|  | T*G interaction |  |  |  |  |  | **2.13** | **0.68** | **0.80** | **3.45** | **0.002** |
| CMJ15% | Group | -197.3 | 136.3 | -464.4 | 69.8 | 0.148 | **-442.9** | **164.0** | **-764.3** | **-121.5** | **0.007** |
| power (W) | Time | **88.3** | **34.7** | **20.3** | **156.2** | **0.011** | 6.39 | 43.8 | -79.5 | 92.3 | 0.884 |
|  | T*G interaction |  |  |  |  |  | **163.8** | **63.8** | **38.8** | **288.7** | **0.010** |
| CMJ25% | Group | -1.00 | 1.02 | -3.01 | 1.00 | 0.327 |  |  |  |  |  |
| height (cm) | Time | 0.46 | 0.37 | -0.26 | 1.17 | 0.212 |  |  |  |  |  |
|  | T*G interaction |  |  |  |  |  |  |  |  |  |  |
| CMJ25% | Group | -149.5 | 142.7 | -429.2 | 130.3 | 0.295 |  |  |  |  |  |
| power (W) | Time | **87.3** | **31.8** | **24.9** | **149.7** | **0.006** |  |  |  |  |  |
|  | T*G interaction |  |  |  |  |  |  |  |  |  |  |
| CMJ50% | Group | -0.90 | 0.79 | -2.44 | 0.65 | 0.256 |  |  |  |  |  |
| height (cm) | Time | 0.37 | 0.33 | -0.27 | 1.01 | 0.256 |  |  |  |  |  |
|  | T*G interaction |  |  |  |  |  |  |  |  |  |  |
| CMJ50% | Group | -180.8 | 120.3 | -416.6 | 55.0 | 0.133 |  |  |  |  |  |
| power (W) | Time | 65.2 | 41.5 | -16.1 | 146.6 | 0.116 |  |  |  |  |  |
|  | T*G interaction |  |  |  |  |  |  |  |  |  |  |

CMJ = counter-movement jump, MVC = maximum voluntary contraction, SJ = squat jump.

**Supplementary table 3.** Changes in body composition analyzed by general equation estimation.

|  |  | Model 1 | | | | | Model 2 | | | | |
| --- | --- | --- | --- | --- | --- | --- | --- | --- | --- | --- | --- |
|  |  | B | SE | 95 % CI Lower | 95 % CI Upper | p-value | B | SE | 95 % CI Lower | 95 % CI Upper | p-value |
| Body | Group | -2.17 | 3.23 | -8.50 | 4.16 | 0.502 |  |  |  |  |  |
| mass (kg) | Time | **0.69** | **0.23** | **0.24** | **1.13** | **0.003** |  |  |  |  |  |
|  | T*G interaction |  |  |  |  |  |  |  |  |  |  |
| Lean mass | Group | -0.57 | 1.64 | -3.79 | 2.66 | 0.730 |  |  |  |  |  |
| (kg) | Time | **0.43** | **0.20** | **0.04** | **0.81** | **0.003** |  |  |  |  |  |
|  | T*G interaction |  |  |  |  |  |  |  |  |  |  |
| Fat mass | Group | -1.52 | 2.24 | -5.91 | 2.87 | 0.497 | -2.73 | 2.50 | -7.63 | 2.16 | 0.273 |
| (kg) | Time | 0.15 | 0.20 | -0.25 | 0.55 | 0.469 | -0.26 | 0.29 | -0.83 | 0.32 | 0.381 |
|  | T*G interaction |  |  |  |  |  | **-0.81** | **0.39** | **0.05** | **1.57** | **0.036** |
| Lower body | Group | -0.28 | 0.90 | -2.04 | 1.47 | 0.751 |  |  |  |  |  |
| lean mass | Time | **0.30** | **0.08** | **0.14** | **0.45** | **<0.001** |  |  |  |  |  |
| (kg) | T*G interaction |  |  |  |  |  |  |  |  |  |  |
| CSA (cm^2^) | Group | -1.64 | 0.85 | -3.30 | 0.02 | 0.053 |  |  |  |  |  |
|  | Time | **1.28** | **0.30** | **0.69** | **1.86** | **<0.001** |  |  |  |  |  |
|  | T*G interaction |  |  |  |  |  |  |  |  |  |  |
| PA (°) | Group | 0.56 | 0.67 | -0.75 | 1.87 | 0.399 |  |  |  |  |  |
|  | Time | 0.00 | 0.25 | -0.49 | 0.50 | 0.994 |  |  |  |  |  |
|  | T*G interaction |  |  |  |  |  |  |  |  |  |  |

CSA = cross section area of m. vastus lateralis, PA = pennation angle of m. vastus lateralis.
